# Altered Mucosal Microbiome Diversity and Disease Severity in Sjögren Syndrome

**DOI:** 10.1038/srep23561

**Published:** 2016-04-18

**Authors:** Cintia S. de Paiva, Dan B. Jones, Michael E. Stern, Fang Bian, Quianta L. Moore, Shani Corbiere, Charles F. Streckfus, Diane S. Hutchinson, Nadim J. Ajami, Joseph F. Petrosino, Stephen C. Pflugfelder

**Affiliations:** 1 Department of Ophthalmology, Baylor College of Medicine, Houston, TX, USA; 2 Biological Sciences, Allergan, Irvine, CA, USA; 3 Sjogren Syndrome Support Group, Houston, TX, USA; 4 Dental Branch, Department of Diagnostic and Biomedical Sciences, University of Texas Health Science Center (UTHSC), Houston, TX, USA; 5 Alkek Center for Metagenomics and Microbiome Research, Department of Molecular Virology and Microbiology, Baylor College of Medicine, Houston, TX, USA

## Abstract

There is mounting evidence that the microbiome has potent immunoregulatory functions. We assessed the effects of intestinal dysbiosis in a model of Sjögren syndrome (SS) by subjecting mice to desiccating stress (DS) and antibiotics (ABX). We characterized the conjunctival, tongue and fecal microbiome profiles of patients with SS. Severity of ocular surface and systemic disease was graded. 16S ribosomal RNA gene sequencing characterized the microbiota. ABX + DS mice had a significantly worse dry eye phenotype compared to controls, a decrease in *Clostridium* and an increase in *Enterobacter, Escherichia/Shigella*, and *Pseudomonas* in stool after ABX + DS for 10 days. Goblet cell density was significantly lower in ABX treated groups compared to controls. Stool from SS subjects had greater relative abundances of *Pseudobutyrivibrio*, *Escherichia/Shigella*, *Blautia*, and *Streptococcus*, while relative abundance of *Bacteroides*, *Parabacteroides*, *Faecalibacterium*, and *Prevotella* was reduced compared to controls. The severity of SS ocular and systemic disease was inversely correlated with microbial diversity. These findings suggest that SS is marked by a dysbiotic intestinal microbiome driven by low relative abundance of commensal bacteria and high relative abundance of potentially pathogenic genera that is associated with worse ocular mucosal disease in a mouse model of SS and in SS patients.

Sjögren syndrome (SS) is one of the most common mucosal autoimmune diseases with a reported prevalence of 0.09–2.7% depending on the diagnostic criteria used^1^. A prevalence of 1% would translate to over 4 million afflicted patients in the United States alone. SS primarily affects the secretory glands and mucosal tissues of the eye and mouth. Both the lacrimal and salivary glands become infiltrated with activated CD4^+^ T cells and B cells^2^,^3^. Ocular surface and oral mucosal disease develops from reduced lubrication, as well as from cytokines produced by activated epithelial cells and infiltrating inflammatory cells^4^,^5^,^6^. Salivary hyposecretion causes difficulty chewing and swallowing dry food, dental caries, and oral candidiasis^7^. Dry eye causes ocular symptoms including irritation, blurred and fluctuating vision and can lead to sight-threatening corneal ulceration in severe cases. Additionally, patients develop fatigue, depression, and inflammation in extraglandular tissues including the brain, lungs, and GI tract^8^. The disease manifestations decrease productivity and quality of life. The quality of life impact of the moderate to severe dry eye that occurs in SS was been reported to be equal to moderate to severe angina^9^. The overall economic burden of dry eye disease in the US is estimated at $5 billion annually due to reduced productivity and the cost of therapies^10^. With only one FDA approved drug for dry eye that is effective in only a subset of patients, there is a pressing need for additional therapies, particularly therapies aimed at fortifying natural immunoregulatory pathways.

The glandular and mucosal immunopathological changes in SS have been thoroughly investigated^3^,^11^. Infiltrating CD4^+^ T cells and B cells replace or destroy secretory acini in the salivary and lacrimal glands. The conjunctiva is infiltrated with dendritic cells (DC) and CD4^+^ T cells. These T cells produce increased levels of IFN-γ and IL-17 that cause ocular surface epithelial disease. IFN-γ causes epithelial apoptosis, goblet cell loss, and increased production of cornified epithelial precursor proteins by the surface epithelium that leads to a poorly wettable skin-like surface^12^,^13^,^14^. IL-17 promotes lymphangiogenesis and stimulates production of matrix metalloproteinases (MMPs) that accelerate desquamation of apical epithelial cells leading to corneal barrier disruption^15^,^16^. The factors inciting this immune based mucosal inflammation are not fully understood^17^. The majority of primary SS patients, independent of race and ethnicity, carry the DQA1*0501 allele^18^,^19^. A polymorphism in interferon response factor 5 (IRF5) has also been found to carry an increased risk for SS^20^. Nevertheless, not all individuals with these genetic risk factors develop disease, and the prevalence of this condition in siblings of patients with SS is no higher than the population in general^21^. This suggests that there are yet to be determined environmental risk factors, particularly those that may increase with age.

Intestinal commensal microbiota limit colonization by enteropathogens and maintain mucosal immune homeostasis throughout the body. Commensal symbionts produce factors that support intestinal barrier function, promote generation of tolerogenic DCs and regulatory T cells (Tregs), and modulate cytokine production by NKT cells. Short chain fatty acids (SCFA), primarily butyryric acid (and to a lesser degree propionic and acetic acid) produced by fermentation of dietary starches by commensal taxa has been found to have potent immunomodulatory activity^22^,^23^.

Evidence suggests that intestinal dysbiosis (microbial imbalance) contributes to the pathogenesis of inflammatory bowel disease (IBD)^24^. Mice with antibiotic induced dysbiosis or those raised in germ-free conditions develop more severe intestinal inflammation in a dextran sodium sulfate (DSS) osmotic stress colitis model due to decreases in SCFA-producing commensal microbiota^25^. Furthermore, frequent antibiotic use during the first year of life was found to increase the risk of developing Crohn’s disease^26^, and increased adherent-invasive *Escherichia coli* or decreased *Faecalibacterium prausnitzii*, a major intestinal butyrate produce, have been associated with disease activity^27^.

We have developed and refined an environmental mouse model of dry eye that recapitulates findings in SS. The observed changes correlate well with human disease and include: corneal barrier disruption, decreased conjunctival goblet cell density, increased CD4^+^ T cell infiltration in the conjunctiva, and increased expression of inflammatory cytokines and T- cell related IFN-γ and IL-17 mRNA transcripts in conjunctiva^28^,^29^,^30^,^31^. This model has been used extensively to screen compounds in pre-clinical settings^30^,^32^,^33^,^34^,^35^,^36^,^37^.

SS affects multiple mucosal tissues and their secretory glands leading to reduced levels of innate antimicrobial factors (e.g. antimicrobial peptides and mucins)^4^,^38^,^39^. We hypothesized that SS is also associated with mucosal microbial dysbiosis that could contribute to the development of autoimmune mucosal inflammation in SS. To test this hypothesis, we evaluated the effects of antibiotic-induced reduction in intestinal microbial diversity on the environmentally desiccated ocular surface in a SS mouse dry model. As a pilot study, we also investigated the ocular, oral, and stool microbiome of patients with SS.

We found that depletion of the intestinal microbiome significantly worsens the ocular surface response to desiccation, and there is significantly altered diversity in the oral and intestinal microbiome in SS that is associated with disease severity.

## Results

### Gut dysbiosis worsens SS-like disease in mice

SS affects multiple mucosal tissues and their supporting glands. Because secretory dysfunction of these glands results in reduced levels of growth factors and innate antimicrobial factors, we hypothesized this could result in mucosal microbial dysbiosis. We evaluated the effects of antibiotic (ABX) treatment on severity of ocular disease in a validated mouse dry eye model with features of SS. This model is induced by pharmacological suppression of glandular tear secretion by cholinergic blockade and exposure to a desiccating stress (DS) environment (drafty and <30% relative humidity). Mice develop severe keratoconjunctivitis sicca with features resembling SS, including corneal barrier disruption, loss of conjunctival goblet cells, and increased expression of inflammatory and T-cell related cytokines in the conjunctiva in response to DS^28^,^29^,^30^,^31^.

**Figure 1:**
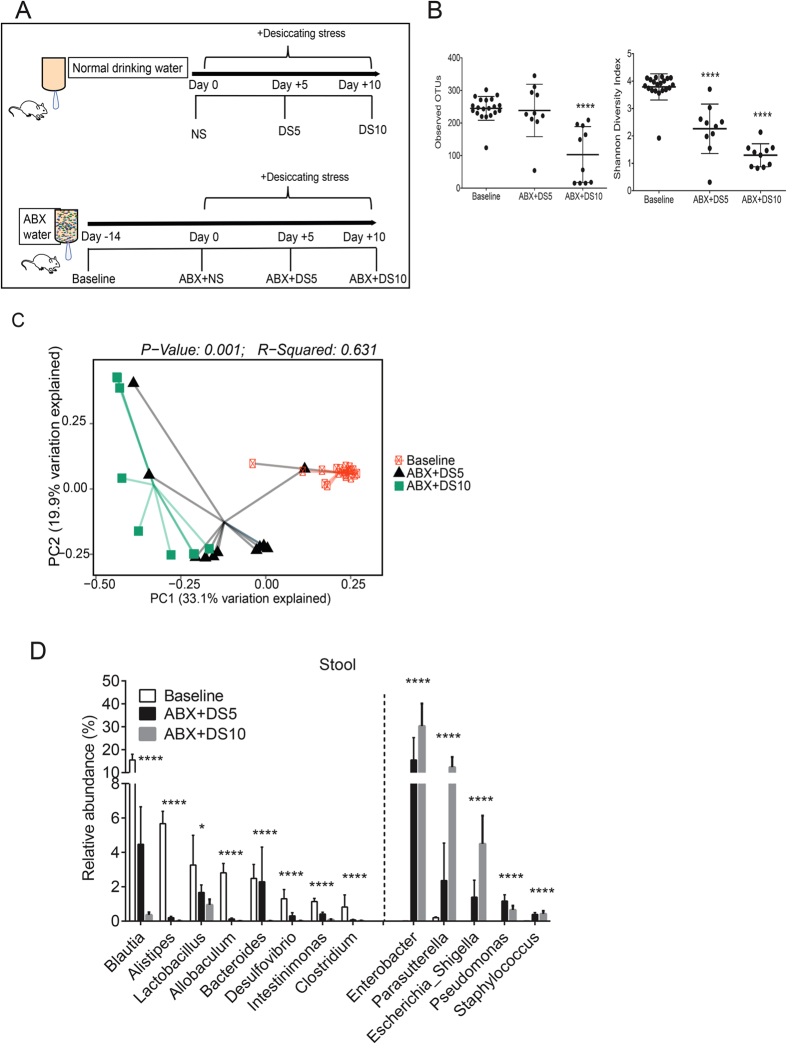
Decreased diversity after antibiotic regimen in mice subjected to desiccating stress. (**A**) Schematic of experimental design. Mice were left non-stressed (NS) or subjected to desiccating stress (DS) for 5 or 10 days (DS5 and DS10) while drinking regular water. A separate group of mice received oral antibiotics (ABX: Ampicillin, Gentamicin, Metronidazole, Neomycin, Vancomycin) in water 14 days prior (baseline) and later were randomized to remain non-stressed (ABX + NS) or to be subjected to desiccating stress for 5 or days while still on ABX water (ABX + DS5 and ABX + DS10, respectively). Thick black arrow indicates duration of water treatment. (**B)** Number of observed operational taxonomic units (OTUs) and Shannon Diversity Index scores in non-stressed mice (NS, baseline) prior to exposure to desiccating stress (DS) with antibiotic cocktail (ABX) for 5 or 10 days (DS5 and DS10, respectively). ****P < 0.0001 compared to baseline group (Kruskall-Wallis test with FDR correction). (**C**) Principal coordinate analysis (PCoA) plot of unweighted UniFrac distances. Each symbol represents an individual sample from baseline and ABX mice subjected to DS for 5 and 10 days. PERMANOVA test, R^2^ = coefficient of determination. (**D**) Comparison of significant relative abundance of different genera among groups. Dotted line divides significant genera that decrease (left) or increase (right) in ABX + DS5 and ABX + DS10 compared to baseline. (Mean ± SEM) *P < 0.05; ****P < 0.0001 (Kruskall-Wallis test with FDR correction).

Our experimental design is presented in Fig. 1A. The effects of our standard DS model on the intestinal microbiome were evaluated by 16S rRNA gene sequencing of stools collected from the same group of mice prior to (non-stressed; NS) and after DS for 5 or 10 days. Mice drank regular water for the duration of the experiment. There was an increase in observed operational taxonomic units (OTUs, P = 0.013, data not shown) and Shannon diversity index at DS10 compared to NS control mice (P = 0.02; Kruskall-Wallis with false-discovery rate (FDR) correction, data not shown). Next we performed UniFrac analysis, which measures dissimilarity between microbial communities accounting for phylogenetic distance and permutational multivariate analysis of variance (PERMANOVA) using distance matrices which detects genetic differences in populations. Clustering was observed among these groups by unweighted UniFrac principal coordinate analysis (P = 0.006, PERMANOVA, data not shown). There was a small shift in the relative abundance of phyla in Proteobacteria in the DS group after 5 days of treatment (4.48 to 7.61%, DS5; P < 0.05; Kruskall-Wallis with FDR correction) and in DS10 compared to NS mice (4.48 vs. 11.94%, P < 0.05; Kruskall-Wallis with FDR correction; data not shown).

We repeated the same protocol using mice that received a cocktail of oral antibiotics (Ampicillin, Gentamicin, Metronidazole, Neomycin, Vancomycin)^40^ for 14 days prior to (baseline) and during the 5 (ABX + DS5) or 10-day (ABX + DS10) exposure to DS. Stool pellets were collected from the same group of mice at baseline and after 5 and 10 days of DS and were processed in the same manner as above. The total duration of antibiotic treatment was 24 days. A significantly lower number of OTUs in ABX + DS10 group compared to baseline was observed and this was accompanied by lower Shannon diversity index (P < 0.0001 for both, Fig. 1B; Kruskall-Wallis with FDR correction). PERMANOVA of unweighted UniFrac metrics demonstrated significant differences in community composition between baseline, ABX + DS5 and ABX + DS10 groups (P = 0.001, R2 = 0.0631, Fig. 1C). We observed that the most abundant phyla showed a progressive and significant decrease in ABX + DS groups (Bacteroidetes: 54.21% vs. 6.51% vs. 0.65%; Firmicutes: 42.63% vs. 15.97% vs. 3.41% in baseline, ABX + DS5 and ABS + DS10, respectively, P < 0.0001 for all; Kruskall-Wallis with FDR correction), while Proteobacteria bloomed (1.66 vs. 59.39 vs. 81.88% baseline, ABX + DS5 and ABS + DS10, respectively, P < 0.001; Kruskall-Wallis with FDR correction). Genera are summarized in Fig 1D. There were significant decreases in *Blautia*, *Alistipes*, *Lactobacillus*, *Allobaculum*, *Bacteroides*, *Desulfovibrio*, *Intestinimonas*, and *Clostridium*, while there were significant increases in *Enterobacter*, *Parasutterella*, *Escherichia/Shigella*, *Pseudomonas*, *Staphylococcus* (P < 0.05 for all, for individual P values see Fig.1D; Kruskall-Wallis with FDR correction).

**Figure 2:**
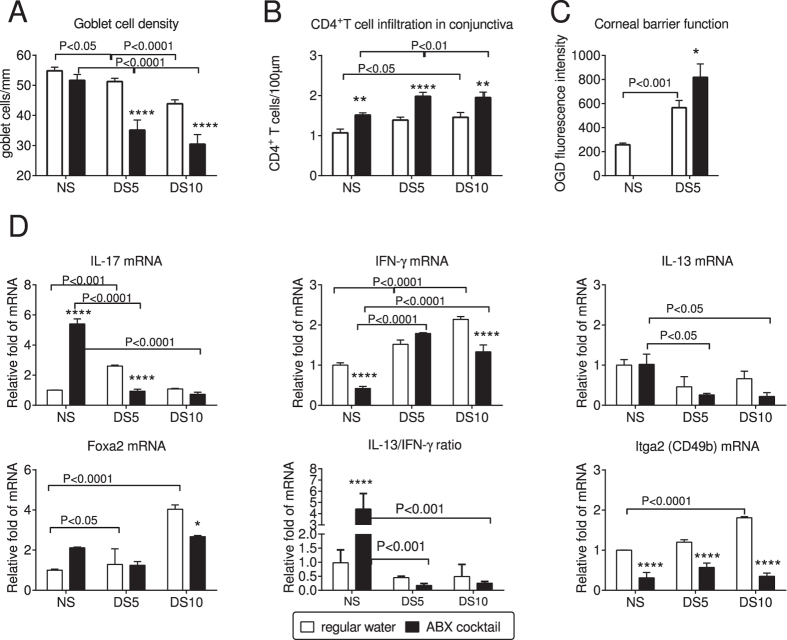
Gut dysbiosis worsens response to desiccating stress and increases production of T-cell related cytokines in conjunctival epithelium. (**A–C**) Goblet cell density (**A**), CD4^+^T cell infiltration (**B**) and corneal barrier function measured by uptake of Oregon-Green Dextran (OGD, **C**) in mice prior to (baseline, NS) and after exposure to desiccating stress with and without antibiotic cocktail (ABX) for 5 or 10 days (DS5 and DS10, respectively). *p < 0.05; **p < 0.01; ***p < 0.001; ****p < 0.0001 water vs. ABX comparisons; Kruskall-Wallis followed by Sidak’s multi-comparisons test. (**D**). Relative fold of expression of IL-17, IFN-γ, IL-13, Foxa2, IL-13/IFN-γ ratio and Integrin alpha 2 (Itga2, CD49b) in conjunctiva from mice prior to (non-stressed, NS) and after exposure to desiccating stress with and without antibiotic cocktail (ABX) for 5 or 10 days (DS5 and DS10, respectively). Data are presented as mean ± SEM of a representative experiment containing 3-4 individual samples/group. Experiment was repeated once with similar results. *p < 0.05; **p < 0.01, ***p < 0.001; ****p < 0.0001 water vs. ABX at each time point, calculated by Kruskall-Wallis followed by Sidak’s multi-comparisons test.

Phenotypically, mice subjected to ABX + DS had greater goblet cell (GC) loss (ABX + DS5 and ABX + DS10, P < 0.001 for both, Kruskall-Wallis with Sidak’s test), increased number of CD4^+^T cells infiltrating the conjunctival epithelium (ABX + DS5 and ABX + DS10, P < 0.001 for both time points; Kruskall-Wallis with Sidak’s test), and greater corneal barrier disruption (ABX + DS5, Fig. 2A–C, P < 0.05; Kruskall-Wallis with Sidak’s test) than mice subjected to DS alone.

We have previously shown that IL-13 is a homeostatic factor maintaining conjunctival GC, while IFN-γ induces conjunctival GC apoptosis and cornification^12^,^41^,^42^. The effects of IL-13 may be due in part to suppression of *Forkhead box A2* (*Foxa2)*, a transcriptional repressor of GC differentiation and mucus biosynthesis, as IL-13-stimulated cultured murine GC had upregulated Muc5ac, Muc2 and resistin-like molecule β (RELMβ) and decreased *Foxa2* gene expression^43^. RELMβ produced by goblet cell maintains epithelial barrier function in the gut and may have similar functions on the ocular surface^44^,^45^. Gene expression analysis of conjunctiva demonstrated that ABX treatment in non-stressed (NS) B6 mice increased IL-17 and decreased IFN-γ mRNA transcripts compared to NS mice that drank normal water (Fig. 2). However, ABX treatment during DS increased IFN-γ mRNA and significantly decreased IL-13 and IL-13/IFN-γ ratios compared to mice that drank ABX but were non-stressed. There was also a decrease in expression of the NK/NKT associated integrin alpha 2 in all ABX treated groups compared to mice that drank regular water.

Taken together, these results demonstrate that desiccating stress alone (regular water) causes a significant increase in Proteobacteria, while oral antibiotic treatment and DS results in extreme changes in the gut microbiota, caused by in a reduction of commensal bacteria and increase in Proteobacteria that was associated with a more severe ocular phenotype. Furthermore, these data provide insight into the molecular mechanisms involved in dysbiosis-induced goblet cell loss.

### Intestinal dysbiosis correlates with disease severity in Sjögren syndrome

After identifying that dysbiosis induced a more severe phenotype in an animal model of SS, we performed a pilot study comparing the microbiome of the conjunctiva, tongue, and stool in subjects with primary SS and healthy control subjects. The conjunctival microbiome in subjects with rosacea that results in a lipid tear deficiency were also evaluated as a comparator group. Demographic features of the study groups are presented in Table 1.

### Ocular surface microbiome

We collected microbiome samples from the inferior conjunctiva of eight normal control subjects, six rosacea patients, and 15 SS patients. Due to the low bacterial biomass on the ocular surface, sequencing yield was lower than anticipated, with an average of 212 mapped 16S sequences per sample. A core microbiome consisting predominantly of Firmicutes, Actinobacteria, Proteobacteria, and Bacteroidetes phyla was found in conjunctival samples from all three groups (control, rosacea, and SS). We did not observe any significant differences in overall composition, richness, or structure of the ocular surface microbiome between the three study groups (Supplemental Fig. 1; Kruskall-Wallis with FDR correction and PERMANOVA).

### Tongue microbiome

The tongue microbiome was compared in ten SS and eleven control subjects (Supplemental Fig. 2). There was no difference in the amount of observed OTUs, but Shannon diversity index score was significantly decreased in SS compared to the other groups (p = 0.0019; Mann-Whitney test) suggesting an uneven distribution of bacterial OTUs (Supplemental Fig. 2A). Significant differences were also observed in the composition of the microbiome as evidenced by distinct clustering by unweighted UniFrac analysis (P = 0.002, R2 = 0.382, Supplemental Fig. 2B; PERMANOVA). The lack of clustering by weighted UniFrac analysis suggests the difference between both groups is primarily driven by low abundant taxa. Nonetheless, we observed an increase of highly abundant *Streptococcus*, in addition to a decrease in *Leptotrichia* and *Fusobacterium* in SS samples compared to controls (P < 0.05 for all, Mann-Whitney test, Supplemental Fig. 2C). Three low abundance genera, *Bergeyella*, *Peptococcus* and *Butyrivibrio*, were also decreased in the SS tongue (Supplemental Fig. 2D).

### Stool microbiome

**Figure 3:**
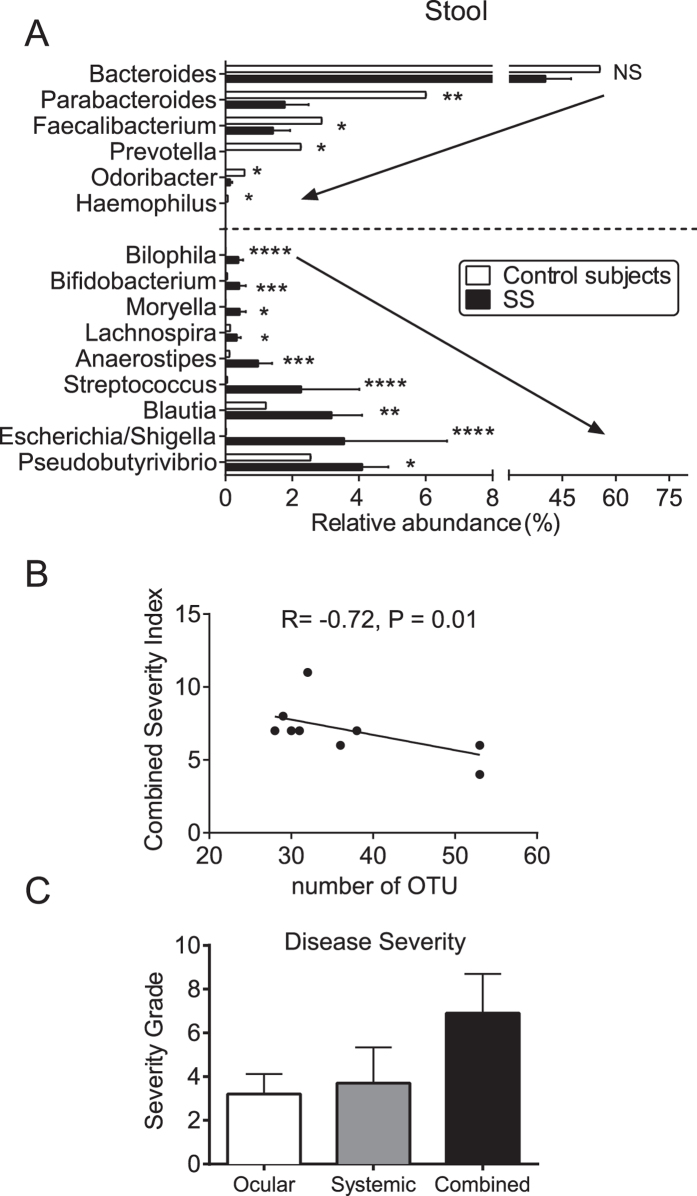
Human stool microbiome in SS. (**A**) Comparison of all significant shifts in the abundance of genera between control subjects and SS patients (Mean ± SEM). Dotted line divides significant genera that decrease (top) or increase (bottom) in SS compared to controls. (**B**) Inverse Pearson’s correlation of combined severity index and number of intestinal OTUs, R = coefficient of correlation (**C**). Ocular severity graded (0–4) Dry Eye Workshop (DEWS) criteria, systemic severity graded (0–33) using unweighted 12-domain ESSDAI (European League against rheumatism (EULAR) Sjögren syndrome disease activity index) and combined ocular and systemic severity (sum of ocular and systemic severity scores). NS = non-significant. *p < 0.05; **p < 0.01, ***p < 0.001; ****p < 0.0001 Mann-Whitney test. NP: not performed.

Stool sequences from ten SS patients were compared to publicly available HMP (Human Microbiome Project) data^46^. The HMP samples were taken from a well-characterized mixed gender healthy control group in which no differences in abundance or diversity of the stool microbiome was found between sexes^47^. The genera with significant between group differences after Mann-Whitney test are presented in Fig. 3A. We observed a greater abundance of *Pseudobutyrivibrio*, *Escherichia/Shigella*, *Blautia*, and *Streptococcus*, while *Bacteroides*, *Parabacteroides*, *Faecalibacterium*, and *Prevotella* were significantly reduced in stool samples from SS individuals (Mann-Whitney test, individual P values are noted in Fig. 3A). There was a 50% decrease in relative abundance of OTUs classified by the NCBI database (≥90% identity) to the high butyrate producer Faecalibacterium prausnitzii by NCBI mapping.

The association between diversity of the stool microbiome and categorical severity of ocular surface, systemic, or combined ocular and systemic disease severity was evaluated. There was a significant inverse correlation between fecal microbial diversity and combined ocular and systemic disease index (r = −0.72. P = 0.01, Fig. 3B), but not systemic disease alone. These severity grades are provided in Fig. 3C.

## Discussion

The intestinal microbiome is one of the more diverse niches in the human body. Members of the Bacteroidetes and Firmicutes phyla dominate the intestinal microbiome, and the microbiome of an individual is more similar to self over time than to others^48^. *Bacteroides* is generally the most abundant genus, but healthy humans exhibit a wide range of *Bacteroides* spp. abundance. *Faecalibacterium prausnitzii* is another abundant microbe in the healthy stool microbiome that is a member of *Clostridium* cluster IV (phylum Firmicutes) and produces SCFA, including butyrate. Decreased abundance of this species has been reported in recurrent IBD^49^. Animal models suggest autoimmunity may be promoted by reduced diversity of the intestinal microbiome with loss of commensal microbes that produce metabolites, such as SCFAs that suppress inflammation by promoting generation of tolerogenic dendritic cells (DCs) and regulatory T cells (Tregs). Reduced diversity may favor emergence of pathogenic bacteria that disrupt the intestinal barrier and stimulate production of inflammatory mediators by mucosal epithelial and resident inflammatory cells in the intestinal lamina propria and mesenteric lymph nodes. This results in the development of pro-inflammatory DCs and autoreactive effector T cells that produce IL-17A or IFN-γ^50^.

The possible role of the microbiome in promoting autoimmunity is just now beginning to be understood, and studies have demonstrated that they can be tissue-specific. For example, in the NOD model of type 1 diabetes, the microbiome was found to inhibit the onset of diabetes in male mice; however, the effects of the microbiome in the SS-like disease that develops in this strain has not been evaluated^51^,^52^. In other models such as experimental autoimmune uveitis and experimental autoimmune encephalomyelitis the microbiome may actively participate in disease development^53^,^54^. Nevertheless, reduced diversity of the stool microbiome induced by ABX has been found to increase severity of the inflammatory response to DSS osmotic stress of the colonic mucosa^25^. Similar to the effects of reduced microbiome diversity on intestinal inflammation, the results of our study showed that antibiotic treatment prior to exposure to DS worsened ocular surface inflammation and epithelial disease (conjunctival goblet loss and corneal barrier disruption) in a mouse model that develops ocular surface disease resembling SS. Antibiotic treatment decreased stool microbiome diversity, including a reduction in *Faecalibacterium*. Desiccating stress in ABX-mice decreased *IL-13*, and increased *IFN-γ* mRNA in the conjunctiva compared to mice that drank ABX but were non-stressed. Consistent with our findings in the SS model, we observed that stool from SS patients had approximately a 50% reduction in the genus *Faecalibacterium*, which includes *Faecalibacterium prausnitzii* one of the predominant butyrate producers in the intestine. There was also a significant increase in potential enteric pathogens, such as *Escherichia/Shigella* and *Enterobacter* in the ABX-treated mice and SS stools. Previous studies have found that antibiotic-treated mice have marked reduction of stool SCFAs^55^ and that supplementation with SCFAs improves inflammation in DSS-induced colitis^25^. A significant decrease in Foxp3^+^Tregs has been noted in colonic lamina propria of GF and ABX-treated B6 mice and conventionalization of GF with feces from normal mice restored the normal frequency of Foxp3^+^Tregs, indicating that signals from microbiota modulate Treg numbers in the gut^56^. Future studies are needed to evaluate if the increased DS induced inflammation in the mouse model and the inverse correlation between stool microbiome diversity in Sjögren syndrome is related to decreased generation of Foxp3^+^Tregs generation.

Conjunctival goblet cells express IFN-γ and IL-3 receptors^41^,^42^ and respond to both cytokines: IL-13 has a role in homeostatic maintenance of GC^41^,^43^ and IFN-γ induces apoptosis, endoplasmic reticulum stress, and unfolded protein response^12^,^14^,^57^. Neutralization of IFN-γ with a topically applied neutralizing antibody during DS increased *IL-13*, decreased *Foxa2* expression, and prevented GC loss^42^. Furthermore, we observed that the ABX cocktail prevented the increase of *integrin alpha 2* transcript, a NK and NKT cell marker. This has potential significance because we have demonstrated that conjunctival resident NKT cells (CD49b + cells) are the major IL-13 producers in the conjunctiva^41^. Intestinal dysbiosis may exacerbate GC loss in dry eye by decreasing the number and altering the cytokine profile of resident NK/NKT cells resulting in decreased Th2 tone.

In this pilot study, we observed distinct differences in the oral and stool microbiome in the SS group compared to controls, characterized by a decreased microbial richness in the mouth and significant differences in certain genera in both mucosal sites. Subjects with the most severe keratoconjunctivitis sicca and combined systemic and ocular disease were found to have the lowest diversity of stool microbiota. It remains to be determined if the observed changes in the mucosal microbiome result from the secretory glandular dysfunction, defective cholinergic signaling with altered gastrointestinal motility, or mucosal disease in SS and/or if the mucosal dysbiosis is a stimulus for development or exacerbation of the autoimmune mucosal and glandular disease immunopathology at these sites.

Although we found differences in the stool and tongue microbiome between control and SS groups, we did not find differences in the conjunctival microbiome. Despite being an exposed mucosa, studies have found very few cultivable bacteria from a swab sample of the conjunctiva^58^. Our study used 16S rRNA sequencing to identify bacterial communities on the ocular surface. We got a good yield of DNA from swab or membrane conjunctival samples, but the majority of sequences mapped to the human genome. On average, the conjunctiva samples had 40 fold fewer reads per sample than the tongue or stool samples. A low biomass sample such as the conjunctiva presents a challenge to correctly identify taxonomic units because the short sequence reads overlap with multiple bacterial and even human sequences. When we included taxa identified in our databases with high probability (>95% identity), we were able to identify a core ocular microbiome composed of the same four phyla in control and SS samples with no difference in abundance between groups. A low abundance ocular surface microbiome of similar composition was also reported by Dr. Russell Van Gelder’s laboratory at the 2015 ARVO meeting (*Doan et al, ARVO meeting abstract, IOVS June 2015, Vol.56, 4067)*. It is possible that differences exist in number and diversity of taxonomic units on the ocular surface between normal and SS eyes, but it will require improved methods to extract and identify the low abundance microbial specific DNA sequences to confirm this.

In summary, we found evidence of oral and intestinal dysbiosis in SS and associations between reduced diversity of intestinal taxa and severity of ocular and systemic disease. This study adds SS to the growing list of autoimmune conditions that are associated with intestinal dysbiosis.

## Material and Methods

### Animals

This research protocol was approved by the Baylor College of Medicine Center for Comparative Medicine, and it conformed to the standards of the Association for Research in Vision and Ophthalmology Statement for the Use of Animals in Ophthalmic and Vision Research. Female C57BL/6 (B6) mice were purchased from Jackson Laboratories (Bar Harbor, ME) and were used at 6-8 weeks of age.

### Standard desiccating stress (DS) model of dry eye

Desiccating stress (DS) was induced in female C57BL/6 mice aged 6–8 weeks by sterile subcutaneous injection of 0.5 mg/mL scopolamine hydrobromide (Sigma-Aldrich, St. Louis, MO) QID into alternating flanks and exposure to a drafty low humidity (<30% relative humidity) environment for 5 or 10 days (DS5 and DS10 respectively) as previously described^15^. Mice subjected to this standard DS model drank regular water.

### Antibiotic treatment and desiccating stress

Six-to-eight week old female C57BL/6 mice (Jackson Labs, Bar Harbor, ME) were treated with a cocktail of broad-spectrum antibiotics [0.5 mg/mL Ampicillin (Dava Pharmaceuticals; Fort lee, NJ), 0.5 mg/mL Gentamicin (Life tech; Grand Islands, NJ), 0.5 mg/mL Metronidazole (Hospira; Lake Forest, IL), 0.5 mg/mL Neomycin (Sparhawk lab; Lenexa, KS), 0.25 mg/mL Vancomycin (Hospira; Lake Forest, IL)] dissolved in drinking water with 5 mg/ml artificial sweetener (Splenda ™, McNeil Nutritionals; Fort Washington, PA) as previously described^40^. Mice drank the ABX cocktail for 14 days prior to and while they were subjected to DS for 5 or 10 days on the beginning of the 15th day.

### Histology and periodic acid-Schiff staining

Right eyes and ocular adnexa were surgically excised (n = 5/group), fixed in 10% formalin, paraffin embedded and were cut into 8-μm sections. Goblet cells in sections were stained with periodic acid-Schiff (PAS) reagent and were examined, photographed and counted with a microscope equipped with a digital camera (Eclipse E400 with a DS-Fi1; Nikon) as previously described^12^.

### Immunohistochemistry

For immunohistochemistry, left eyes and adnexa of mice at each time point (n = 5) were excised, embedded in optimal cutting temperature (OCT compound; VWR, Suwanee, GA), and flash frozen in liquid nitrogen. Sagittal 8-μm sections were cut with a cryostat (HM 500; Micron, Waldorf, Germany), placed on glass slides and stored at −80 °C. The number of CD4^+^ T cells in the conjunctival epithelia was counted in cryosections stained with rat-anti mouse CD4 (clone H129.9, 10 μg/mL, BD Bioscience, San Diego, CA) as previously described^12^.

### Measurement of corneal permeability

Corneal epithelial permeability to Oregon Green Dextran (OGD; 70,000 molecular weight; Invitrogen, Eugene, OR) was assessed by instilling 0.5 μL of OGD onto the ocular surface one minute before euthanasia, as previously described^15^. Corneas were rinsed with PBS and photographed under fluorescence excitation at 470 nm. The severity of corneal OGD staining was graded in digital images in the 2 mm central zone of each cornea by 2 masked observers, using the NIS Elements software (Nikon, Melville, NY). Six to eight mice (12–16 eyes) per each group were examined in two different sets of experiments.

### RNA isolation and Real time PCR

Total RNA from conjunctiva or the corneal epithelium was extracted using a QIAGEN RNeasy Plus Micro RNA isolation kit (Qiagen) following the manufacturer’s protocol. Conjunctiva was surgically excised. One sample equaled the tissue pooled from both eyes of each animal. After isolation, the concentration of RNA was measured and cDNA was synthesized using the Ready-To-Go™ You-Prime First-Strand kit (GE Healthcare) as previously described^15^. Real time PCR was performed using specific Taqman probes for IL-17A (*Il17a*, Mm0043918_m1), IFN-γ (*Ifn-γ*, Mm00801778_m1), IL-13 (*Il13*, Mm99999190_m1), Foxa2 (*Foxa2*, Mm00839704_mH), Integrin α2 (*Itga2 or DX5*, Mm00434371_m1) (Taqman Universal PCR Master Mix AmpErase UNG) in a commercial thermocycling system (StepOnePlus™ Real-Time PCR System, Applied Biosystems), according to the manufacturer’s recommendations. The beta-2 microglobulin (β2 m) (*B2m*) (Mm00437762_m1) gene was used as an endogenous reference for each reaction. The results of quantitative PCR were analyzed by the comparative C_t_ method in which the target of change = 2^−^ΔΔ^Ct^ and were normalized by the C_t_ value of β2 m and the mean C_t_ of relative mRNA level in the untreated naïve group. Gene expression of 3–4 mice per group per time point in two independent experiments with a total of 6–8 mice was determined.

### Statistical Analysis for mouse samples

All experiments were repeated at least two times. After completion of all experiments, data were averaged, and graphs were generated. Sample size calculation was performed with StateMate Software version 2.0 (GraphPad Inc., San Diego, CA), based on preliminary data.

The data consisting of two groups or more groups were analyzed by non-parametric Kruskall Wallis followed by Sidak’s multiple comparison test. P ≤ 0.05 was considered statistically significant. These tests were performed using GraphPad Prism 6.0 software (GraphPad Software, Inc., San Diego, CA, USA).

### Human subjects

The study was conducted in accordance with the Declaration of Helsinki, and the Baylor College of Medicine Institutional Review Board approved the protocol and informed consent form prior to study initiation. Informed consent was obtained from all patients prior the study. Patients with SS were recruited from the multispecialty SS clinic at Baylor College of Medicine (BCM). All SS patients had a complete ocular, oral, and rheumatologic evaluation, including panel of serum autoantibodies and met proposed American College of Rheumatology diagnostic criteria for SS^59^ . Subjects with rosacea had at least two facial features accompanied by meibomian gland disease and unstable tear film. Control conjunctival and tongue samples were obtained from healthy subjects with no signs of symptoms of dry eye, dry mouth or oral disease. Stool samples from healthy subjects were taken from a cohort of subjects from Houston, TX who were included in the Human Microbiome Project^46^. The Dry Eye Workshop (DEWS) criteria were used for categorical grading of ocular severity 0–4 and the unweighted European League against rheumatism (EULAR) Sjögren syndrome disease activity index (ESSDAI) was used for grading severity of systemic disease 0–33^60^,^61^. The combined severity was the sum of the ocular and systemic severity scores and the unweighted ESSDAI score was used to give similar weight to the ocular and systemic disease.

### Sampling of mucosal microbiome

Ocular microbiome was sampled using an EyePrim device (OpiaTech, Paris, France) that applies a sterile Supor 450 membrane against the inferior conjunctival fornix. Tongue microbiome was sampled using a Catch-All™ Specimen Collection swab (Epicentrec Biotechnologies, Madison, WI, USA) rubbed over a 1 cm^2^ area on the dorsal tongue for 5 seconds. Ocular and oral samples were placed into MO-BIO 0.1 mm glass bead tubes, (MO-BIO, Carlsbad, CA) containing 500 μL solution SW1 and immediately frozen at −80 °C. Stool was collected in a commode collection container (Fisher scientific, Waltham, MA, USA) and immediately placed in a Styrofoam container containing cool packs, sealed and delivered to the clinic within 60 minutes where it was placed in a −80 °C freezer until the DNA was extracted.

### Sequence analysis

#### Ocular microbiome

Variable regions 1–3 (v1-3) of the 16S rRNA gene were amplified using previously described primers for conjunctival samples^48^. Concentration and purity of PCR products were determined by PicoGreen (Invitrogen, Carlsbad, CA) and Agilent Bioanalyzer 2100 DNA 1000 chip (Agilent Technologies), respectively. Amplicons were pooled in equimolar concentrations and sequencing was performed on the 454 platform (Roche) according to the manufacturers’ instructions.

#### Tongue and stool microbiome

Bacterial genomic DNA from tongue and stool samples was extracted using the PowerSoil DNA Isolation Kit (MO BIO Laboratories). The 16S rDNA V4 region was amplified by PCR and sequenced in the MiSeq platform (Illumina) using the 2 × 250 bp paired-end protocol yielding pair-end reads that overlap almost completely. The primers used for amplification contain adapters for MiSeq sequencing and dual-index barcodes so that the PCR products may be pooled and sequenced directly^62^.

16S rRNA gene sequences were assigned into OTUs at a similarity cutoff value of 97% using UPARSE and the SILVA Database^63^,^64^. Abundances were recovered by mapping the demultiplexed reads to the UPARSE OTUs. Human tongue, human stool, and mouse stool samples were rarefied to 10,000 reads/sample. A custom script constructed an OTU table from the output files generated in the previous two steps, and then used to calculate alpha-diversity, beta-diversity^65^ and provide taxonomic summaries. Kruskall-Wallis non-parametric test was used to test for overall significance between groups followed by False-Discovery Rate (FDR) to account for multiple comparisons. Mann-Whitney test was used to evaluate comparisons between two groups. PERMANOVA test was applied to determine if microbial composition and structure differed between groups.

## Additional Information

**How to cite this article**: de Paiva, C. S. *et al*. Altered Mucosal Microbiome Diversity and Disease Severity in Sjögren Syndrome. *Sci. Rep*. **6**, 23561; doi: 10.1038/srep23561 (2016).

## Supplementary Material

Supplementary Information

## Figures and Tables

**Table 1:** Demographic Information for study subjects.

	Conjunctiva	Tongue	Stool
	Number, Mean age ± SD (years), Gender F/M		
Sjögren Syndrome	N = 1563 ± 1015 F/0 M	N = 1059 ± 1410 F/0 M	N = 1059 ± 1410 F/0 M
Rosacea	N = 657 ± 203 F/3 M	NP	NP
Control	N = 857 ± 288 F/0 M	N = 1138 ± 157 F/2 M	N = 45*27 ± 519 F/26 M

^*^Cohort of normal subjects from Houston, TX who were included in the Human Microbiome Project. F=female; M=male; NP=not performed; SD=standard deviaton.
