# Supplementary material for: Altered Mucosal Microbiome Diversity and Disease Severity in Sjögren Syndrome
Source: Sci Rep. 2016 Apr 18;6:23561. doi: 10.1038/srep23561 (PMC4834578; doi:10.1038/srep23561)
Supplement: Supplementary Information [file srep23561-s1.doc]

**Altered Mucosal Microbiome Diversity and Disease Severity in Sjögren Syndrome**

Cintia S. de Paiva, Dan B. Jones, Michael E. Stern, Fang Bian, Quianta L. Moore, Shani Corbiere, Charles F. Streckfus, Diane S. Hutchinson, Nadim J. Ajami, Joseph F. Petrosino, Stephen C. Pflugfelder


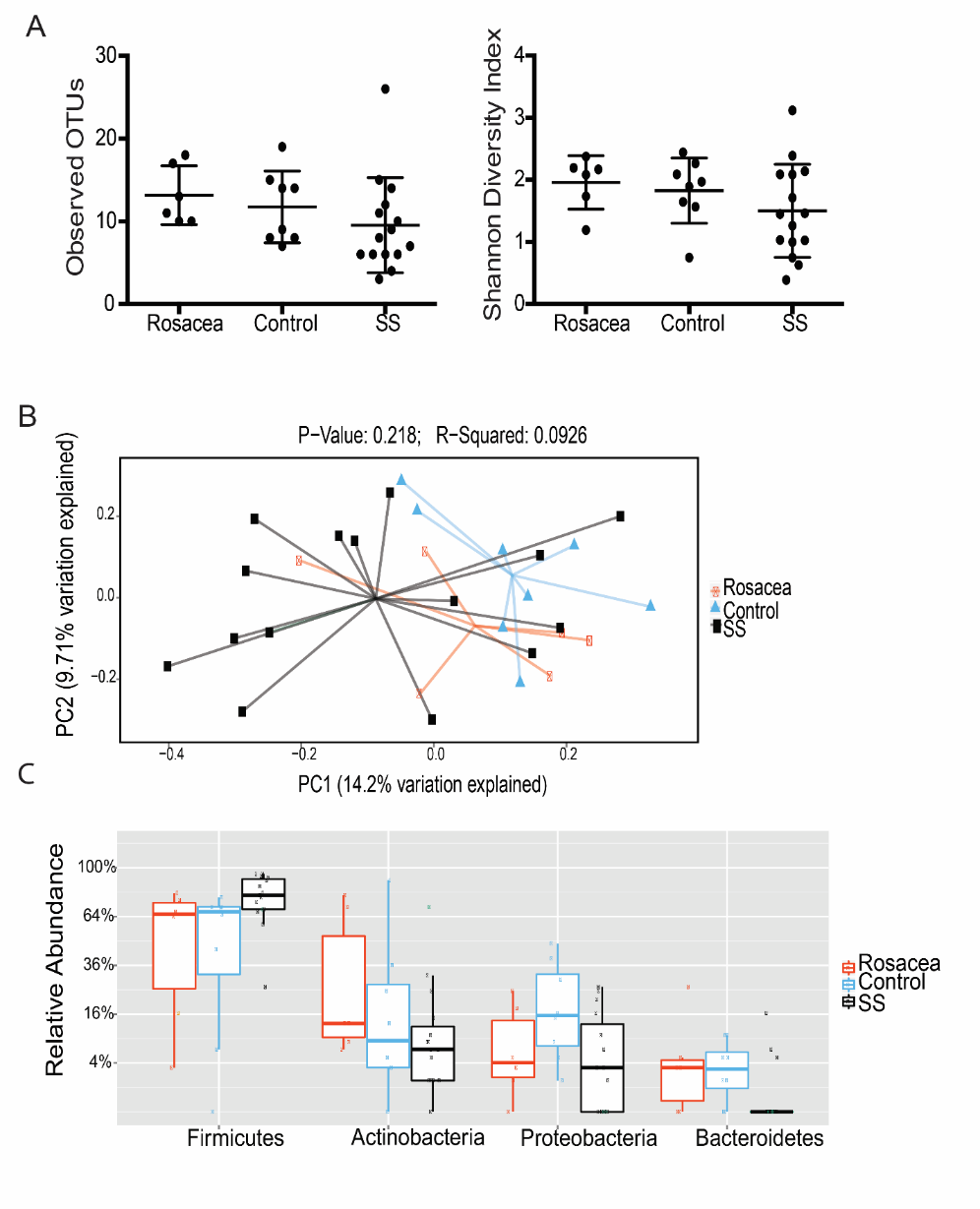


**Supplemental Figure 1. Ocular microbiome.**

A. Number of observed operational taxonomic units (OTUs) and estimates of bacterial diversity as assessed by the Shannon index.

B Principal coordinate (PC) analysis plot of unweighted UniFrac distances. Each symbol represents an individual sample from control, ocular rosacea, and SS patients.

C. Comparison of the relative abundance of different phyla among control, rosacea, and SS patients. (Mean±SEM). No statistical differences were found.


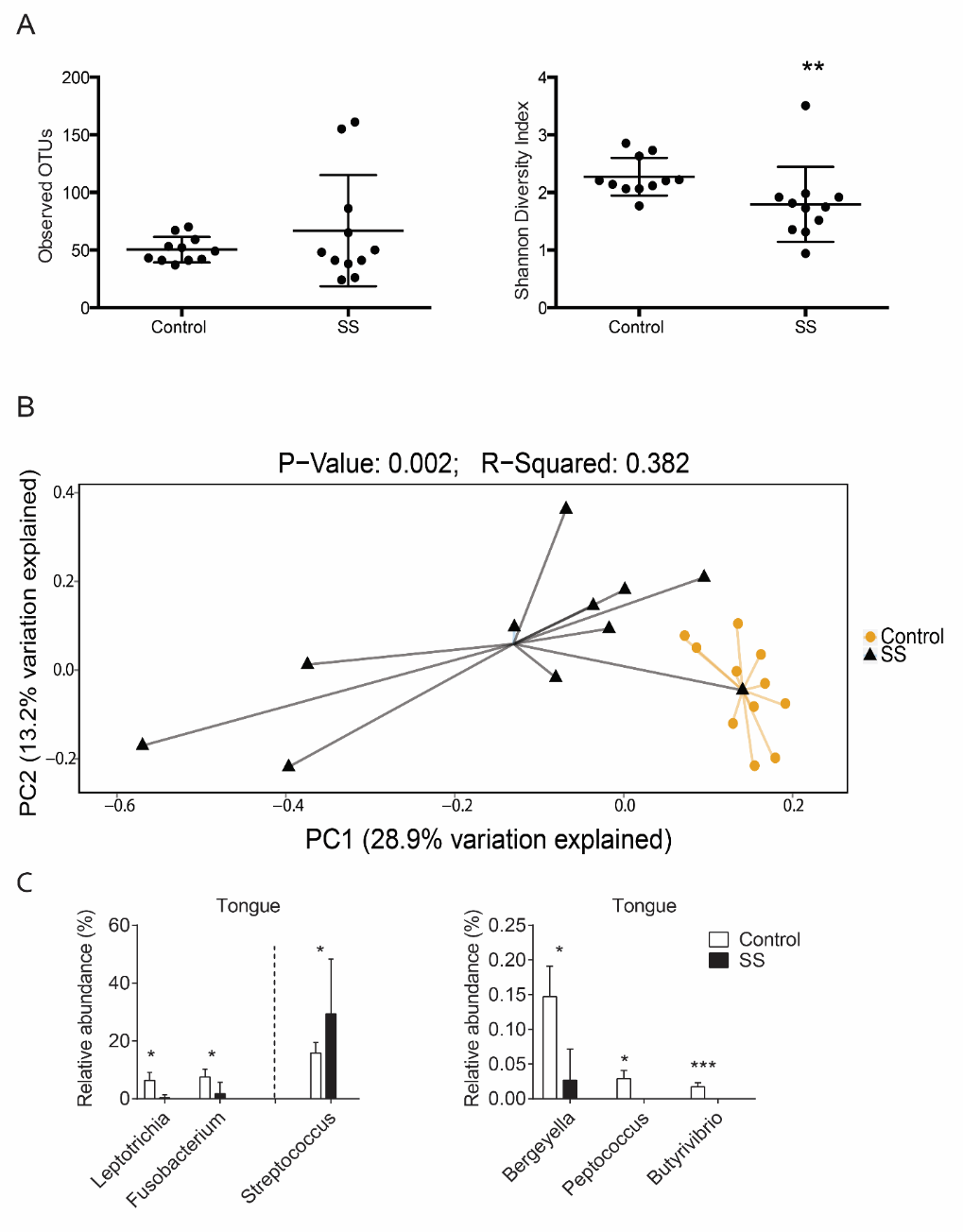


**Supplemental Fig. 2. Tongue microbiome in SS**

A. Number of observed OTUs and estimates of bacterial diversity as assessed by the Shannon index. B. Principal component (PC) analysis plot of unweighted UniFrac distances sequenced at an even sampling depth across samples. Each symbol represents the microbiota of a single stool-associated community from control subjects, and SS patients. C. Comparison of the relative high (left) and low (right) abundance at taxonomic units at the genus level among control subjects and SS patients. (Mean±SEM). Dotted line indicates relative high abundance genera that increased in SS relative to controls. *p<0.05;**p<0.01, ***P<0.001; ****P<0.0001 Mann-Whitney test
